# Efficacy of China-made praziquantel for treatment of Schistosomiasis haematobium in Africa: A randomized controlled trial

**DOI:** 10.1371/journal.pntd.0007238

**Published:** 2019-04-10

**Authors:** Xin-Yao Wang, Jian He, Saleh Juma, Fatma Kabole, Jia-gang Guo, Jian-Rong Dai, Wei Li, Kun Yang

**Affiliations:** 1 Jiangsu Institute of Parasitic Diseases, Wuxi, Jiangsu, China; 2 Key Laboratory on Technology for Parasitic Disease Prevention and Control, Ministry of Health; Jiangsu Provincial Key Laboratory on the Molecular Biology of Parasites, Wuxi, Jiangsu, China; 3 College of Medicine, Jiangnan University, Wuxi, Jiangsu, China; 4 Ministry of Health of Zanzibar, Zanzibar, United Republic of Tanzania; 5 Department of control of Neglected Tropical Diseases, World Health Organization, Geneva, Switzerland; Swiss Tropical and Public Health Institute, SWITZERLAND

## Abstract

**Background:**

In the roadmap on the neglected tropical diseases (NTD) the World Health Organization (WHO) aims at attaining at least 75% coverage of preventive chemotherapy in pre-school and school-age children by 2020. A randomized controlled trial was used to compare the effectiveness of praziquantel in treating *Schistosoma haematobium* in Africa using two different sources for the drug, Merck Limited Partnership (KgaA), Germany and Nanjing Pharmaceutical Factory (NPF), China.

**Methods:**

More than 6,000 participants testing positive for *S*. *haematobium* infection were enrolled from three villages (shehias) situated in the northern, middle and southern part of Pemba Island, Zanzibar. Applying criteria of inclusion and exclusion, resulted in a study population of 152 people (84 males, 68 females). A randomized controlled trial was conducted assigning participants to either praziquantel from NPF or Merck KGaA. After one month, the cure rate of *S*. *haematobium* and adverse events were compared to evaluate effectiveness. The ratio of male to female, the ratio of light/high infection intensity, and the average value of age were calculated between the two drug manufacturers. Chi-squared test and T-test were used for consistency analysis.

**Results:**

Out of the total of 73 cases receiving praziquantel from NPF, the cure rate achieved was 97.3% (73/75), while the 74 cases receiving the drug from Merck KgaA reached a similar cure rate (96.1% or 74/77). There was no significant difference between the two outcomes (χ^2^ = 0.003, P = 0.956). Among the 75 patients treat, only one (a 16-years old female student), who had received the drug made in China had slight adverse reactions manifested as dizziness, headache and abdominal pain.

**Conclusion:**

The efficacy of China-made praziquantel does not differ significantly from praziquantel made by Merck KGaA in Germany.

**Trial registration:**

ClinicalTrials.gov, NCT03133832

## Author summary

Schistosomiasis is a neglected tropical disease (NTD) with praziquantel the only effective drug available. It is estimated that about 800 million people are currently at risk for this disease with more than 200 million people infected. A large drug donation partnership between Merck, Darmstadt, Germany and the World Health Organization supports widespread, successful preventive chemotherapy programs, but the donated drugs does not meet the entire need, particularly not in Africa. The primary aim of our study was to evaluate the efficacy of China-made praziquantel to explore whether the drug could complement preventive chemotherapy programs in Africa. A randomized controlled trial was conducted assigning participants to two groups, one receiving praziquantel from NPF, the other from Merck KGaA. After one month, the cure rate of *S*. *haematobium* and were compared to evaluate effectiveness and document potential adverse events. The outcome showed that the two sources of praziquantel that did not differ significantly from each other. China-made praziquantel can thus be trusted to complement the current use of the drug from Merck KGaA in Africa making it possible to enlarge drug provision programs.

## Introduction

In tropical and subtropical regions, schistosomiasis remains as an important public health problem. It is estimated about 800 million people are at risk of schistosomiasis infection and more than 200 million people are constantly infected [1, 2]. The trematode digenetic parasites in the family Schistosomatidae (phylum Platyhelminthes) infect a wide range of vertebrates. Five major species of the genus *Schistosoma* are major medical importance: *Schistosoma mansoni*, *S*. *japonicum*, *S*. *haematobium*, *S*. *intercalatum* and *S*. *mekongi* [3, 4]. *S*. *haematobium* is distributed in different area of Africa including Zanzibar and the Middle East [5] where it infects the genito-urinary tract of humans causing urinary schistosomiasis, whose many complication include the bladder cancer [6, 7].

Praziquantel is an anthelmintic drug whose complete principle of action remains to be unraveled, but it is known that a main effect is paralysis of the function of the worm's sucker[8–11]. It is highly effective and remains the drug of choice for the treatment of all forms of schistosomiasis infection [12], and is also active against a broad range of parasitic helminths, including clonorchiasis, opisthorchiasis, tapeworm infections, cysticercosis and hydatid disease [13]. Although safe and generally without serious side effects, praziquantel can still cause poor coordination, abdominal pain, vomiting, headache and allergic reactions. While it may be used in women during pregnancy for the second trimester, it is not recommended for the mother when breastfeeding [14].

The strategy of WHO to eliminate schistosomiasis involves a large-scale treatment for affected populations through periodic, targeted treatment of school-children with praziquantel [15]. In 2008, Merck KgaA, Darmstadt, Germany started a program donating 20 million tablets (600mg) annually, which is still ongoing [16].

The increased use of the drug is attributable to many factors, including improved availability of donated praziquantel, essentially from Merck, which has led to some countries implementing large-scale schistosomiasis control programmes and scale-up of praziquantel treatment [17]. The global target of WHO in the roadmap on neglected tropical diseases (NTD) is to attain at least 75% coverage of preventive chemotherapy in pre-school and school-age children by 2020 [18]. Praziquantel has been used to treat schistosomiasis for a long time in China and the experience demonstrates that preventive chemotherapy (i.e., large-scale treatment without individual diagnosis) with high coverage significantly impacts infection indices and even reduces transmission [19].

In 2014, WHO signed a tripartite memorandum of understanding (MOU) with China and Zanzibar, paving the way for the start of a pilot schistosomiasis elimination programme in Zanzibar. The objectives are to control and eliminated schistosomiasis based on the experience of schistosomiasis control in China. The project on Pemba Island is China^’^s first aid project focusing on schistosomiasis control. The early stage of the project was to carry out schistosomiasis prevention and control work in three villages (shihias), named Mtangani, Kiuyu and Wingwi, using snail control, population investigation and treatment [20–23]. In a follow-up step, we proposed an open-label, randomized trial to evaluate the comparative efficacy China-made praziquantel in the treatment people infected with *S*. *haematobium* in Africa. This paper describes this approach.

## Methods

### Ethics statement

The field studies did not involve endangered or protected species. All subject enrollment has been signed the informed consent of this study. For all participants who were not adult, a parent or guardian provided informed consent on their behalf. The China-made praziquantel used was certified by the Zanzibar foods and Drugs Board (ZFDB). The study was approved by the local government of Pemba Island. In addition, the Ethics Review Committee of Zanzibar approved all studies described here (ZAMREC/002/MAY/014). The trial was registered with ClinicalTrials.gov, number NCT03133832.

### Study design and participants

Zanzibar is composed of two sister islands, Unguja and Pemba, situated off the eastern coast of Tanzania mainland between latitudes 4 and 5 degrees South [24]. Zanzibar is endemic for schistosomiasis haematobium. The intermediate host is *Bulinus globosus* [25, 26]. In 2011, a survey of 24 schools showed that the infection in Unguja and Pemba rates were 8% (0–38%) and 15% (1–43%), respectively [26].

A randomized controlled trial was used for this study [27]. Three shehias, Mtangani, Kiuyu and Wingwi, situated in northern, central and southern Pemba, respectively, were selected as study area. Three pilot areas proceeded schistosomiasis control in the early stages of assistance. A series of meetings was held at the shehias and their schools to explain the objectives, procedures, and potential risks of the study. More than 6,000 participants were originally enrolled for the study. Applying criteria of inclusion and exclusion (see below), resulted in a final study population of 152 people (84 males, 68 females).

### Randomization and masking

People were enrolled randomly into two groups: group A received praziquantel (batch number: 20170303) from Nanjing Pharmaceutical Factory Co Ltd (NPF), Nanjing, China and group B received praziquantel (batch number: M50812) from Merck Limited Partnership (Merck KgaA), Darmstadt; Germany donated from WHO for the treatment of schistosomiasis in Africa.

The dose was 40mg/kg body weight of drug given in a single oral dose according to the instructions of the manufacturers. The Merck KgaA praziquantel was larger than that made by NPF. Nothing less than half a tablet was given. Thus, if the fraction calculated ended up as an amount less than that, half a tablet was still given [28–30]. The staff of the NTD office, Ministry of Health, Zanzibar would instruct the drug distributers after confirming the treatment allocation from the randomization sequence that had been generated by computer. The NTD staff and participants was unmasked to the treatment assignment, but the laboratory technicians were masked to samples examination throughout the study.

### Procedures

Inclusion criteria were the following: people permanently living on the island including residents and students of ages between 5 and 60 years who had been shown to be infected by *S*. *haematobium* as confirmed by urine test (worm eggs detected). Exclusion criteria were the following: people refusing chemotherapy of praziquantel; women pregnant or lactating at the time of the study; people with serious adverse drug reactions; patients with severe heart, liver or kidney problems; those with a history of mental illness; and patients who for various reasons did not take medication on time.

The NTD staff recorded the exact time of drug ingestion. Participants were observed for 2 hours after taking the drug to see adverse events and were continually observed at home by the assistance of local public health community center (PHCU)staff. They followed up on the medications every day to see adverse events. If anybody was vomiting within 2 hours of drug ingestion, a second dose was given.

Urine was collected and tested in April 2017. Every participant provided a fresh urine sample used to detect the presence of *S*. *haematobium*. The NTD staff subjected all potential participants to physical examination and eligibility check. We used a 10 ml syringe to extract 10 ml and filtered the urine, repeatedly shaking the cup to include eggs that that has a tendency to collect at the bottom. The filter device was prepared according to WHO recommendations with the filter taken out and placed on a glass slide before microscopy. Only participants found positive and otherwise meeting the criteria were included in the study.

After one month, follow-up was conducted with urine were collected and tested in the same way again. Signs and symptom indicating adverse effects, inter-current illness or abdominal pain was recorded for the preceding month. Quality control measure was used for inter-observation variability, and technician retested a random selection of 10% of slides in the laboratory. At end of the study, all participants who still excreted *S*. *haematobium* eggs (i.e., who had not been cured) were again treated with praziquantel.

### Outcome

The primary outcome was a comparison of the cure rates after either having received praziquantel from NPF or Merck KgaA. The secondary outcome was the rate of severe adverse events.

### Statistical analysis

Serial report forms were used to collect the data from the participants. Epi Info (Centers for Disease Control and Prevention), SPSS 20.0 (International Business Machines Corporation) and PASS 16 (NCSS) were used for data entry and analysis.

The sample size was calculated by cross-sectional survey, 95% confidence interval (CI) (Zα/2 = 1.96), 5% margin of error and design effect of 2.5. According to previous study, the infection rate of schistosomiasis in Pemba was about 6.0% and following the function of $\text{N}=Z_{1-\alpha /2}^{2}\left(1-p\right)/\varepsilon^{2}p$ [31]. Therefore, the sample size calculation formula required approximately 6,019 cases. The outcome of power was 0.99975, which met the requirements. For the estimation of sample size in the early stage, we set the accuracy to 10% of the expected incidence rate to estimate the sample size.

In this study, *S*. *heamatobium* infection intensity was determined as light (1–49 egg/10ml urine) and heavy (≥50 eggs/10ml urine) [32]. The ratio of male to female, the ratio of light infection intensity to heavy, and the average value of age was firstly calculated between the NPF and Merck KgaA praziquantel groups, Chi-squared test and T-test were used to analysis the consistency.

A two-sided of Chi-squared test was used to compare the cure rate of positive and adverse reactions between different groups. The size of the test or α level was set to 5%, with 95% CI, with exact binomial estimates when necessary. The number of eggs and logarithmic transformation was in a skewed state, so the median value was used to analysis the change of infection intensity between the NPF and Merck KgaA praziquantel groups after praziquantel treatment.

## Results

### The basic information

**Fig 1:**
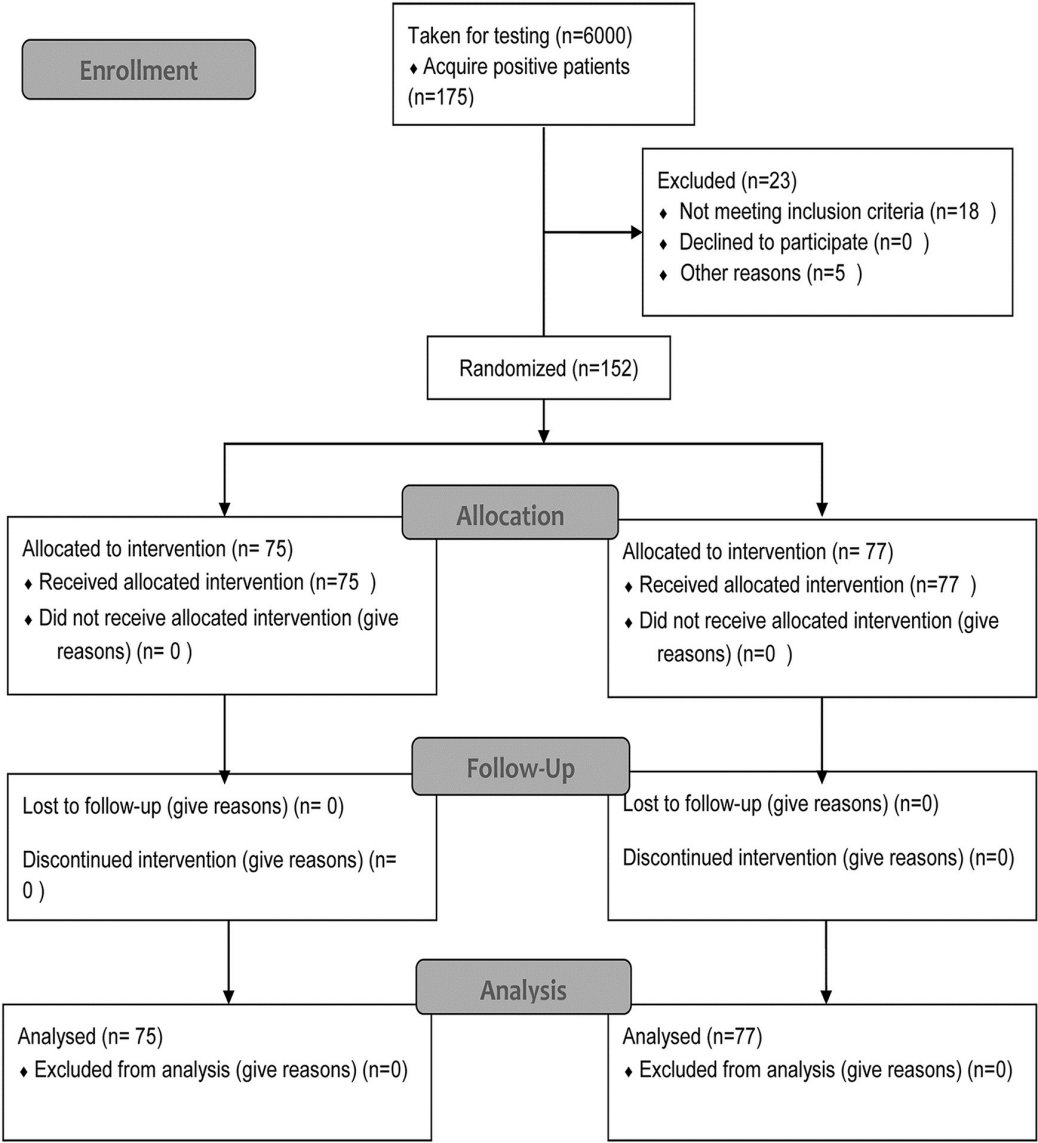
Flow diagram.

A total of 6000 people were enrolled from three shehias in Pemba Island Zanzibar, Urine samples were collected and tested in April 2017 (Fig 1). According to the criteria of inclusion and exclusion, 152 people (84 males, 68 females) were included consisting of 45 participants from Mtangani, 76 participants from Kiuyu and 31 participants from Wingwi. Participants were randomly assigned to study groups and in May 2017, 75 people received NPF praziquantel (males: 37, females: 38) and 77 Merck KgaA praziquantel (males: 47, females: 30) (Table 1). There was no significant difference of age in the two groups (t = -0.424, P = 0.672) with a median of 14 for both Merck KGaA and NPF. There was no significant difference of the number of eggs in the two groups (t = -1.130, P = 0.261) with the median value in Merck KGaA and NPF 8 and 9, respectively. There was good consistency with respect to age and gender for *S*. *haematobium* infection and intensity between the two groups.

**Table 1:** The characteristics of participants in three Shehias in Pemba island, Zanzibar.

Shehia	Drug type	Participant no. (female pro- portion %)	Number of males	Males (% pro- portion)	Mean age (SD)	Median age (interquartile range)	Light infection* (% proportion)	Heavy infection** % proportion)
Mtangani	NPF	21(9.4%)	9	43%	13.4 (2.4)	13.0 (12.0–16.0)	17(81%)	4(19%)
	Merck KgaA	24(14.6%)	15	63%	14.2 (2.4)	13.0 (13.0–17.0)	22(92%)	2(8%)
Wingwi	NPF	16(5.3%)	5	31%	31.0 (19.3)	27.0 (14.0–46.8)	11(69%)	5(31%)
	Merck KgaA	15(6.4%)	6	40%	28.0 (19.6)	27.0 (12.5–45.0)	11(73%)	4(27%)
Kiyuyu	NPF	38(23.6%)	23	61%	17.1 (12.1)	13.5 (10.0–17.0)	31(82%)	7(18%)
	Merck KgaA	38(26.7%)	26	68%	16.3 (12.0)	14.0 (11.0–19.8)	33(87%)	5(13%)
Total	NPF	75(37.5%)	37	49%	18.9 (13.7)	14.0 (11.0–17.0)	59(79%)	16(21%)
	Merck KgaA	77(47.6%)	47	61%	18.0 (14.3)	14(5–70)	66(86%)	11(14%)

*1–49 eggs per milliliter of urine;

**≥50 eggs per milliliter of urine.

### Treatment effects

Seventy-three of the 75 cases receiving the NPF praziquantel were all negative translating into a cure rate of 97.3%. With regard to the Merck KgaA praziquantel, treatment the cure rate was 96.1% (74/77). There was no significant difference in the cure rate between the two groups (χ2 = 0.003, P = 0.956).

In Mtangani, 20 were people tested negative out of the 21 who had received the NPF praziquantel (cure rate = 95.2%), while 21 of the 24 receiving the Merck KgaA praziquantel were cured translating into a rate = 87.5%. There was no statistically significant difference (χ^2^ = 0.038, P = 0.845). In Wingwi, 16 patients received praziquantel from NPF and 15 were negative (cure rate = 93.8%). With regard to Merck KgaA praziquantel, all 15 treated patients were negative, so the cure rate was 100.0%. Also here, there was no statistical significant differences between the two groups (χ^2^ = 0.016, P = 0.900). In Kiuyu, finally, there were 38 people in both groups and they were all cured, so the cure rate was again 100.0% in two groups.

As seen in Tables 2 and 3, there was no statistical difference with respect to gender (male: χ^2^ = 0.027, P = 0.869; female: χ^2^ = 0.001, P = 0.977) or the degree of infection (light: χ^2^ = 0.0003, P = 0.986; heavy: χ^2^ = 0.001, P = 0.973).

**Table 2:** Results of urine sample testing before and after treatment of NPF and Merck KgaA praziquantel with respect to gender.

Drug	Gender	Positive before treatment (no.)	Negative after treatment (no.)	Negative rate after treatment (% proportion)	Results of chi-square test (p-value)
NPF praziquantel	Male	37	37	100.00%	0.869
	Female	38	36	94.74%	
Merck KgaA praziquantel	Male	47	45	95.74%	0.977
	Female	30	29	96.67%	

**Table 3:** Results of urine sample testing before and after treatment of NPF and Merck KgaA praziquantel with respect to the degree of infection.

Degree of infection	Drug	Positive before treatment (no.)	Negative after treatment (no.)	Negative rate after treatment (% proportion)	Results of chi-square test (p-value)
Light degree	NPF praziquantel	28	27	96.43%	0.986
	Merck KgaA praziquantel	66	64	96.97%	
Heavy degree	NPF praziquantel	9	8	88.89%	0.973
	Merck KgaA praziquantel	11	10	90.91%	

One month after treatment, only two cases were found in the NPF group; the number of eggs was 1 and 6, respectively, while three cases appeared in the Merck KGaA group with 3, 12 and 20 eggs, respectively. The final egg reduction rate was thus 99.8% (4285/4292) in the NPF group and 98.7% (2620/2655) in the Merck KGaA group.

### Side effects

The side effects included dizziness, headache, nausea, anorexia, abdominal pain, diarrhea, vomiting, body weakness, cough, itchy skin and skin rash. Among the 152 patients, only one 16-year old female student experienced slight adverse reactions, manifested as dizziness and headache of receiving the NPF product. No other adverse effects occurred.

## Discussion

Praziquantel is a heterocyclic isoquinolinazine derivative synthesized in 1972 by the two firms E. Merck and Bayer Pharmaceuticals [33]. Outside Germany, the drug was first synthesized by Shin Poong PharmaceuticaL Co. in South Korea and in 1977 also in a Chinese company in 1977 and subsequently was used in clinical practice there in 1978. Due to a high curative effect, low side effects and high safety, praziquantel is not only used for clinical treatment on a large scale of various stages of schistosomiasis in China, but also used for prevention of schistosomiasis [34, 35]. Although *S*. *haematobium* is widespread in Africa, no large amounts of China-made praziquantel made by NPF has been used on a large scale. Most praziquantel used in Africa comes is from Merck KgaA through the WHO donation mentioned above [36–38]. However, the actual demand over the last 5 years was about 400 million tablets, which above what can be met by the donation. It is thus important to find another source of effective praziquantel that is not expensive [16, 39, 40].

This study investigated the feasibility of China-made praziquantel to complement what is currently available. Non-inferiority margins limit the allowable range of clinically acceptable medicines and comparator drugs. From the perspective of clinical efficacy, and against the background of previous test results, it must be repeatedly demonstrated that a drug is superior to these margins. Sometimes, a cost-benefit analysis may be necessary. However, this study did not investigate the efficacy of praziquantel *per se*, but used traditional statistical methods to compare the differences in efficacy between the two products to prove the possibility of using Chinese praziquantel for the treatment and prevention of schistosomiasis haematobium. For non-inferiority margins, relevant studies will be conducted again in the future.

This study may be limited in that the design of testing only relatively few people from three shehias. However, this was done to serve as a reflection of the overall situation on Pemba Island. The studies proved to be a strong indication there was no difference between the NPF praziquantel and that made by Merck KgaA. There was also no stratified study of Shehias in this study. In the results, the cure rates of different shehias were compared, and then the overall cure rates were compared, just to observe whether there were statistical differences in different shehias, and then make an overall comparison. In terms of drug prices, China-made praziquantel prices are cheaper, which is conducive to large-scale application in Africa [41–44].

Although praziquantel is effective in the treatment of schistosomiasis, side effects have been reported clinically and in the field [45, 46]. We recorded only one adverse effect, which could be due to differences in drug manufacture [47]. However, this occurrence was to minor, and a large-scale trail would be needed to rule this out or put it on a better footing. The fact that the common side effects, mainly reactions from the neuromuscular, digestive, or cardiovascular system sometimes due to allergy, are generally light and disappear in a short time, makes a comparison difficult without reverting to large-scale trails. Even severe reactions may occur in individual cases, such as syncope, ataxia, angina pectoris and severe arrhythmia, might be easier to record but they are very rare [48]. Finally, what makes the study of side effects difficult is that they may be related to the dose, route, time, dosage form, physical properties of the drug, and individual differences in the drug administration [33, 49, 50]. Through studying of other researches, China-made praziquantel had also good effectiveness to treat schistosomiasis haematobium [51].

There were some confounding factors, such as re-infection after treatment. Since only 152 positive patients were collected in the first year of the project, the sample size may not be sufficiently large to study that. On the other hand, with one month period after treatment, there is hardly time for new infections to reach the maturity needed to produce eggs. There was no equivalence study, so in this study we used a traditional comparison of two drugs to prove the possibility of China-made praziquantel treatment of schistosomiasis.

In conclusion, this study indicates that China-made praziquantel has good efficacy and minor side effects. China-made praziquantel can be used to treat *S*. *haematobium* infections in Africa on a large scale.

## Supporting information

S1 Checklist CONSORT checklist. (DOC)

S1 CONSORT Flow diagram. (DOC)
