# Supplementary material for: Efficacy of China-made praziquantel for treatment of Schistosomiasis haematobium in Africa: A randomized controlled trial
Source: PLoS Negl Trop Dis. 2019 Apr 10;13(4):e0007238. doi: 10.1371/journal.pntd.0007238 (PMC6476521; doi:10.1371/journal.pntd.0007238)
Supplement: S1 CONSORT — (DOC) [file pntd.0007238.s002.doc]

**
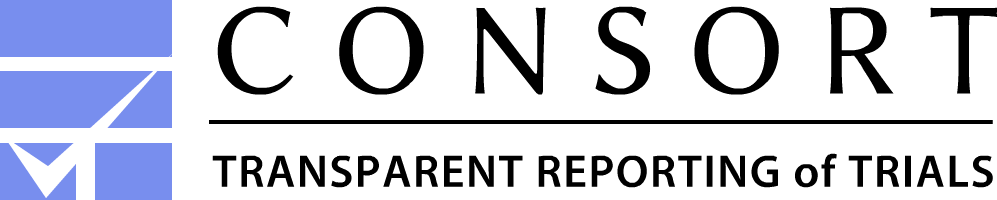
**

**CONSORT 2010 Flow Diagram**

**Allocation**

**Analysis**

**Follow-Up**

**Enrollment**

Taken for testing (n=6000)

♦ Acquire positive patients (n=175)

Excluded (n=23)

♦  Not meeting inclusion criteria (n=18 )

♦  Declined to participate (n=0 )

♦  Other reasons (n=5 )

Analysed (n= 75)
♦ Excluded from analysis (give reasons) (n=0)

Lost to follow-up (give reasons) (n= 0)

Discontinued intervention (give reasons) (n= 0 )

Allocated to intervention (n= 75)

♦ Received allocated intervention (n=75 )

♦ Did not receive allocated intervention (give reasons) (n= 0 )

Lost to follow-up (give reasons) (n=0)

Discontinued intervention (give reasons) (n=0)

Allocated to intervention (n= 77)

♦ Received allocated intervention (n=77 )

♦ Did not receive allocated intervention (give reasons) (n=0 )

Analysed (n=77)
♦ Excluded from analysis (give reasons) (n=0)

Randomized (n=152)
